# Disorganized attachment and identity dissociation: *FKBP5* CATT as molecular moderator

**DOI:** 10.1016/j.psychres.2026.117079

**Published:** 2026-03-04

**Authors:** Leonhard Kratzer, Keith Gaynor, Hans Knoblauch, Abigail Powers, Seyma Katrinli, Vasiliki Michopoulos, Negar Fani, Charles F. Gillespie, Tanja Jovanovic, Kerry J. Ressler, Alicia K. Smith, Bertram Müller-Myhsok, Stefan Tschöke

**Affiliations:** a Department of Psychotraumatology, Clinic St. Irmingard, Prien am Chiemsee, Germany; b School of Psychology, University College Dublin, Dublin, Ireland; c Clinic for Psychiatry and Psychotherapy I (Weissenau), Ulm University, Ulm, Germany; d Centre for Psychiatry Suedwuerttemberg, Ravensburg, Germany; e Department of Psychiatry and Behavioral Sciences, Emory University School of Medicine, Atlanta, GA, USA; f Department of Gynecology and Obstetrics, Emory University School of Medicine, Atlanta, GA, USA; g Department of Psychiatry and Behavioral Neurosciences, Wayne State University School of Medicine, Detroit, MI, USA; h Department of Psychiatry, McLean Hospital, Harvard Medical School, Belmont, MA, USA; i Research Group Statistical Genetics, Max Planck Institute of Psychiatry, Munich, Germany

**Keywords:** Identity dissociation, Childhood maltreatment, Gene-environment interaction, *FKBP5 haplotypes*, Attachment

## Abstract

**Background:**

Childhood maltreatment and *FKBP5* CATT haplotypes increase identity dissociation risk. While maltreatment often fosters disorganized attachment, the specific role of the *FKBP*5 CATT haplotype within this developmental pathway remains unknown.

**Methods:**

In 310 trauma-exposed adults, we assessed childhood maltreatment, disorganized attachment, and identity dissociation. *FKBP*5 CATT haplotype status was dichotomized (present/absent). Regression models were estimated to examine the pathway Childhood Maltreatment → Disorganized Attachment → Identity Dissociation, comparing two competing hypotheses: 1) The Formation Hypothesis (Maltreatment × CATT → Disorganization), and 2) The Translation Hypothesis (Disorganization × CATT → Identity Dissociation). Model fit was compared using BIC, and simple slopes were examined.

**Results:**

Disorganized attachment predicted identity dissociation (β = 0.143, *z* = 3.83, *p* < .001), and childhood maltreatment predicted disorganization (β = 0.007, *z* = 4.46, *p* < .001). The Formation hypothesis was not supported, as the Maltreatment × CATT interaction did not predict disorganization (β = −0.004, *z* = −1.18, *p* = .24). However, the Translation hypothesis was supported by three pieces of evidence: a significant Disorganization × CATT interaction predicting identity dissociation (β = 0.133, *z* = 2.22, *p* = .027), model fit (ΔBIC = 265.3), and simple slopes showing a strong effect of disorganization on identity dissociation for CATT carriers (β = 0.15, *p* < .001; +16.5 % per unit) but not for non-carriers (β = 0.02, *p* = .65).

**Conclusions:**

The *FKBP*5 CATT haplotype does not promote disorganized attachment but amplifies the translation of disorganized attachment into identity dissociation. This result highlights a genetically influenced failure of identity development following disorganized attachment.

## 1. Introduction

Childhood maltreatment is a risk factor for the development of severe psychopathological phenomena later in life, including dissociative symptoms ranging from depersonalization to identity dissociation (Kratzer et al., 2024; Vonderlin et al., 2018). The latter, characterized by experiences of discrete self-states and a fragmented sense of self, has long been understood as a failure of integration in response to overwhelming stress and represents a particularly severe outcome linked to long-term personality difficulties and dissociative disorders (Briere et al., 2005; Dalenberg et al., 2012; Janet, 1889; Luyten et al., 2020). At the same time, marked heterogeneity in outcomes suggests that trauma exposure alone is insufficient to explain (identity) dissociation (Lynn et al., 2022, 2019).

The link between childhood maltreatment and dissociation is well-established (Vonderlin et al., 2018), and dissociation appears to act as a transdiagnostic psychological process increasing the risk of a range of diagnostic presentations beyond dissociative disorders. For example, this holds for Borderline Personality Disorder (Kurt and Çakır, 2025; Luyten et al., 2020; Peng et al., 2021), depression (Shim et al., 2024; Wrobel et al., 2022), positive and negative symptoms of psychosis (Alameda et al., 2020; Bloomfield et al., 2021; Gaynor et al., 2024; Grady et al., 2025, 2024; Onyeama et al., 2024), somatic distress among individuals with severe mental illness (Orme et al., 2021), suicidal behavior (Szeifert et al., 2025), and post-traumatic stress disorder (Kratzer et al., 2018; Muller et al., 2012; Zvi and Rachimi, 2024). This highlights the need to integrate developmental, cognitive, and neuro-biological mechanisms to understand how childhood maltreatment impedes identity and how this develops a diverse range of psychopathological presentations (Kearney and Lanius, 2022).

Recent research on attachment and self-structure emphasizes identity-related processes as a central pathway from early adversity to psychopathology. For example, Baroncelli and colleagues (2025) show that childhood trauma and attachment difficulties are closely tied to a lack of sense of coherent self. Similarly, the Memory and Identity Theory of ICD-11 Complex PTSD (CPTSD) proposes that traumatic memories and negative identities (e.g., experiencing onseself as defeated or worthless) are central mechanisms linking trauma to CPTSD symptomatology (Hyland et al., 2023; Lahav et al., 2025), which is closely tied to dissociative symptomatology (Hyland et al., 2024a, 2020; Mohammadi et al., 2024). Empirical evidence shows that fragmented trauma memories and disrupted identity processes are core features of CPTSD (Hyland et al., 2025; 2024b; Kearney and Lanius, 2024).

Attachment theory provides a complementary, transdiagnostic framework. Developmental models suggest that when caregivers are abusive, frightening, or insensitive, children experience an irresolvable inner conflict off light and approach, leading to disorganized attachment (Liotti, 2006, 2004). Meta-analytic evidence confirms that childhood maltreatment profoundly disrupts attachment organization and predisposes to disorganized attachment (Cyr et al., 2010). Maltreatment, whether overtly abusive or in the form of neglect and emotional unavailability, impairs the infant’s capacity to develop a coherent strategy for seeking security, which may lead to disintegrative processes, a fragmented self, and dissociation (Ogawa et al., 1997). Building on this evidence, recent conceptualizations of “attachment trauma” postulate that childhood maltreatment triggers distinct pathogenic processes, such as traumatic disintegration, detachment, and dissociation, which may contribute to identity disturbances (Farina et al., 2019; Farina and Imperatori, 2023; Farina and Schimmenti, 2025).

Importanty, the sequential pathway *Childhood maltreatment* → *Attachment Disorganization* → *Dissociation* provides a mechanistic link between these bodies of research. The diverse literature, despite its often diagnosis-specific approach, is unified in describing a process whereby disintegrative and dissociative mechanisms, initially protective within a traumatic and disorganized attachment environment, ultimately impede the integration of experience necessary for a coherent sense of self. This resulting lack of mental organization and basic self-disorder creates a transdiagnostic vulnerability for a variety of severe psychological sequelae (Huang et al., 2020; Kearney and Lanius, 2022; Lanius et al., 2011; Ong et al., 2025). Thus, a fundamental failure of integration acts as a transdiagnostic mechanism, creating vulnerability for severe psychopathological symptoms across diagnostic categories, one of the most severe sequelae being identity dissociation (Loewenstein and Brand, 2023).

Neurobiological models complement these perspectives, showing that chronic maltreatment alters brain structures and networks crucial for emotion regulation, autobiographical memory, and self-representation (Teicher et al., 2016). Taken together, attachment-based, clinical, and neurobiological evidence converges on a multi-level pathway: childhood maltreatment disrupts attachment, memory integration, and self-development, thereby increasing risk for complex trauma-related psychopathology, including identity dissociation (Burback et al., 2024; Hyland et al., 2023; Kearney and Lanius, 2022).

Despite growing evidence for these pathways, it remains unclear why some individuals with maltreatment histories, attachment disorganization, and identity-related vulnerability develop severe identity fragmentation while others do not. Genetic variation regulating stress-response plasticity is a likely contributor (Raffagnato et al., 2025). The glucocorticoid receptor is an important modulator of the hypothalamic-pituitary-adrenal (HPA) axis. In the HPA axis, FK506-binding protein 5 (*FKBP5*) is involved in modulating glucocorticoid receptor sensitivity in response to stress. Proteins have complex three-dimensional structures (conformation), which are frequently stabilized by so-called Chaperones. Co-chaperones, in turn, are molecules that support and regulate chaperones in this process. They do not directly participate in protein folding (Binder et al., 2004). The *FKBP5* gene encodes such a co-chaperone. Common haplotypes of the *FKBP5* gene are formed by four single nucleotide polymorphisms (SNPs) *(rs9296158, rs3800373, rs1360780, and rs9470080).*

Specific risk alleles, such as the CATT haplotype, are associated with altered receptor sensitivity and prolonged cortisol responses via epigenetic mechanisms (Zannas et al., 2015). These variants may render neurobiological systems more plastic but also more vulnerable, amplifying the risk of stress-induced psychiatric disorders (Halldorsdottir et al., 2019; Malekpour et al., 2023). Recent work indicates that *FKBP5* CATT and maltreatment interact to predict identity dissociation (Kratzer et al., 2026, in press; Moser et al., 2025).

However, the precise point at which *FKBP5* moderates the trauma-to-dissociation pathway remains unknown. We consider two hypotheses:

1. Formation Hypothesis: *FKBP5* variation may sensitize the attachment system to maltreatment, increasing the likelihood that early adversity produces disorganized attachment and identity-related disturbances.
2. Translation Hypothesis: Alternatively, *FKBP5* variation may impair the neural capacity to restore integration once attachment disorganization and identity vulnerability are established, amplifying the progression from disorganization to clinical identity dissociation.

Clarifying this distinction is critical for understanding the etiology of identity dissociation. Our research defines a transdiagnostic endophenotype, i.e., a key vulnerability, and shows how the interaction between childhood maltreatment, disorganized attachment, and the *FKBP5* gene leads to the biological and psychological basis of identity dissociation. We

- replicate the link of disorganized attachment and identity dissociation (H1; *disorganized attachment* → *identity dissociation*),
- replicate the link of childhood maltreatment and disorganized attachment (H2; *childhood maltreatment* → *disorganized attachment*),
- and test whether *FKBP5* CATT moderates the *formation* of disorganized attachment (H3a formation hypothesis; *Childhood Maltreatment x CATT* → *Disorganized Attachment*) or the *progression* from disorganization to identity dissociation (H3b translational hypothesis; *Disorganized Attachment* *x* *CATT* → identity dissociation).

## 2. Methods

### 2.1. Ethics

All study procedures were approved by the Institutional Review Boards of Emory University School of Medicine and Grady Memorial Hospital. This study was performed in accordance with the ethical principles outlined in the Declaration of Helsinki and its later amendments (World Medical Association, 2024). Data handling and processing conformed to all applicable Health Insurance Portability and Accountability Act rules. Data was de-identified to preserve both anonymity and confidentiality. Furthermore, this study was conducted under the research exception provisions of the Privacy Rule, 45 CFR 164.514(e), and was therefore exempt from institutional review board informed consent requirements. No new human participants which had not been previously included in prior studies were recruited for this study.

### 2.3. Participants and procedure

The study sample comprised *N* = 310 participants drawn from a large study on the impact of trauma-related risk factors for psychopathology in a high risk, trauma-exposed urban population of Black Americans in Atlanta, USA, with a sample size of *N* = 11,524 (Gillespie et al., 2009; Gluck et al., 2021). Inclusion criteria for the current study were age ≥ 18 years, the experience of at least one traumatic event, the ability to provide informed consent, assessments of childhood maltreatment using the Childhood Trauma Questionnaire (CTQ) (Bernstein and Fink, 1998), disorganized attachment using the Adult Attachment Prototype Questionnaire (AAPQ) (Westen et al., 2006), and dissociation using the Multiscale Dissociation Inventory (MDI) (Briere, 2002), as well as genetic data on the presence of the CATT haplotype. The only exclusion criterion in the overall study was psychiatric hospitalization within 30 days of enrollment. Participants were recruited between 2011 and 2016 at Grady Memorial Hospital, Atlanta, Georgia, USA. The sample consisted predominantly of a trauma-exposed urban population of Black Americans (*n* = 280 women [90.3 %], *n* = 30 men [9.7 %]). The mean age was 40.81 years (*SD* = 11.70; min = 20; max = 65). Additional sociodemographic characteristics (education, relationship status, employment) are presented in Table 1.

### 2.4. Measures

Dissociative symptoms were assessed using the MDI (Briere, 2002), a comprehensive self-report measure that captures various dimensions of dissociation, including depersonalization, derealization, memory disturbance, and identity dissociation. However, for the current study, we focused specifically on the Identity Dissociation subscale as our primary dependent variable. This decision was driven by recent findings indicating that the *FKBP5* CATT haplotype is uniquely associated with identity dissociation, i.e. the most severe form of compartmentalization, but not with other dissociative dimensions such as detachment (Moser et al., 2025). The subscale identity dissociation assesses dissociative identity alterations and uses items like “Having different people inside of you with different names”, “Feeling like there was more than one person inside of you”, and “Switching back and forth between different personalities”. The MDI comprises 30 items, each rated on a 5-point Likert scale ranging from 1 (“never”) to 5 (“very often”).

Childhood maltreatment exposure was assessed using the CTQ (Bernstein and Fink, 1998). This 28-item self-report instrument evaluates five types of childhood maltreatment: emotional and physical neglect as well as emotional, sexual, and physical abuse. Items are rated on a 5-point Likert scale, ranging from 1 (“never trueȁ) to 5 (“very often true”). Subscale scores were summed to create a total childhood maltreatment score, which was centered, and used as a continuous predictor for this study.

Disorganized attachment was assessed with the Disorganized subscale of the AAPQ (Westen et al., 2006). The AAPQ is a clinician-rated instrument that indexes four attachment prototypes: secure, dismissive, preoccupied, and disorganized. For each prototype, clinicians provide a global prototype rating (1 = “no match” to 5 = “very strong match”) and, if the prototype is at least moderately characteristic (rating ≥ 2), they additionally rate specific follow-up items on a 5-point scale (1 = “untrue” to 5 = “very true”). In the present study, we used only the disorganized prototype rating but not its follow-up indicators. This choice was theoretically driven as disorganized attachment is the specific pattern theorized to result from frightening or abusive caregiving (Liotti, 2006) and is the core attachment pattern linked mechanistically to the failure of integration and the development of severe dissociation (Ogawa et al., 1997). While the other insecure prototypes may co-occur or inter-correlate with disorganized attachment, the disorganized pattern provides the most direct test of the attachment-based pathway to identity fragmentation. Higher scores on the disorganized prototype scale reflect more disorganized attachment representations. Participants completed the MDI and CTQ assessments during research visits with study staff which were also used for AAPQ ratings.

### 2.5. Genetics

Genotyping focused on four *FKBP5* single nucleotide polymorphisms (SNPs; rs3800373, rs9296158, rs1360780, rs9470080) that define the *FKBP5* CATT haplotype. Genotypes were obtained using Illumina OmniQuad 1 M and OmniExpress arrays. Genotype data processing and quality control followed the RICOPILI pipeline developed by the Psychiatric Genomics Consortium PTSD Workgroup (PGC-PTSD; Nievergelt et al., 2019). Quality control steps included removal of SNPs with call rates < 95 %, monomorphic SNPs, and SNPs with > 2 % differences in missingness between PTSD cases and controls. Samples were excluded if they showed call rates < 98 %, extreme inbreeding coefficients (f_het < −0.2 or > 0.2), or discrepancies between reported and genetically inferred sex (based on X-chromosome SNPs). SNPs that deviated from Hardy-Weinberg equilibrium in controls (*p* < 1 × 10^−6^) were also removed.

Relatedness between individuals was assessed using identity-by-state estimates in PLINK 1.9, and one individual from each pair with $\hat{\pi }>0.2$ was excluded, prioritizing retention of PTSD cases when possible. To account for population stratification, the first 10 principal components (PCs) of genetic ancestry were derived via multidimensional scaling (MDS). Prior to MDS, QCed data were filtered for minor allele frequency (MAF > 0.05), minimum genotyping frequency of 2 %, and linkage disequilibrium pruning (r^2^ < 0.2). These ancestry components (PC1-PC10) were included as covariates in all association analyses.

Phasing of QCed genotype data on chromosome 6 was performed using SHAPEIT with the 1000 Genomes Phase 3 reference panel (Auton et al., 2015; Delaneau et al., 2019), allowing assignment of alleles to their respective homologous chromosomes. For each individual, the alleles at the four *FKBP5* SNPs were then combined to construct the *FKBP5* CATT haplotype. The resulting haplotype count was dichotomized (0 = no CATT haplotype; 1 = ≥ 1 CATT haplotype) to maximize statistical power for the interaction term, yielding a binary genetic moderator variable (non-carrier vs. carrier).

### 2.6. Statistical analyses

**Fig. 1.**
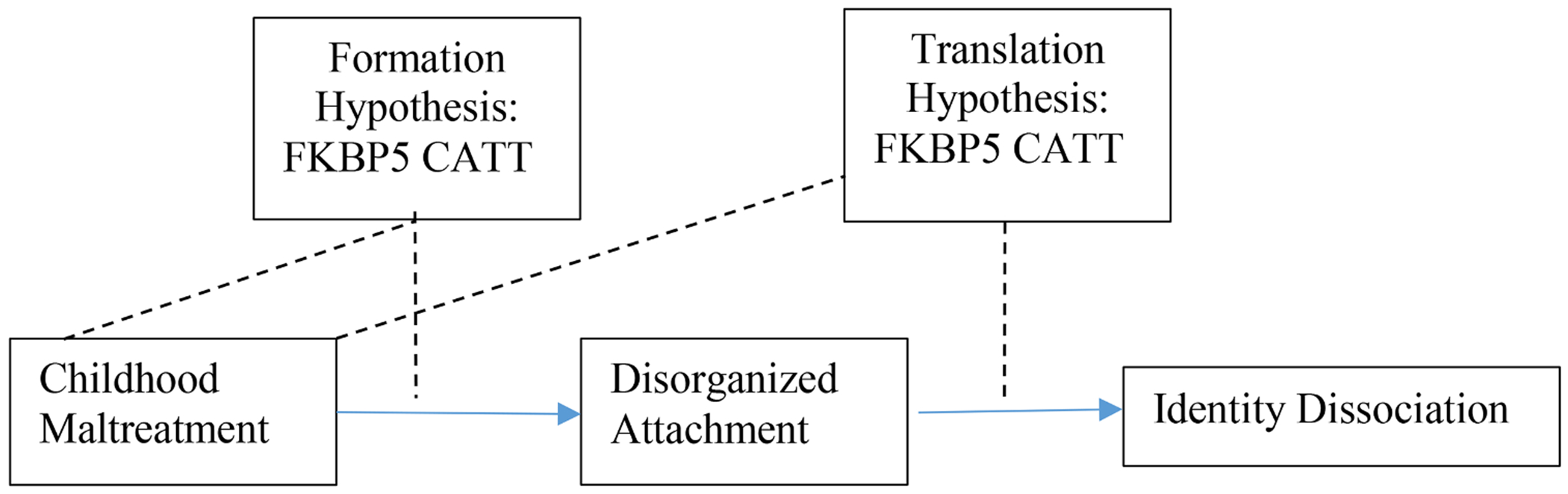
Competing Hypotheses for the Moderating Role of FKBP5 CATT Haplotype: The Formation Hypothesis and the Translation Hypothesis.

The analytic strategy comprised two main steps. First, we tested the basic attachment-based pathway from childhood maltreatment to identity dissociation. Second, building on prior evidence that CTQ × *FKBP5* CATT predicts identity dissociation in this sample, we examined where along this pathway *FKBP5* CATT exerts its moderating effect (see Fig. 1).

All primary models were estimated as generalized linear models (GLMs) with a Gamma distribution and a log link: log(*E*[*Y_i_*]) = *η_i_*. This specification accommodates the strictly positive, right-skewed distributions of identity dissociation and disorganized attachment, allowing for multiplicative effects on the mean outcome. HC3 robust standard errors and corresponding robust *z*-tests were computed for all coefficients. Link-function adequacy was checked by comparing Gamma models with log, identity, and inverse links, and ordinary least squares (OLS) regression of log-transformed identity dissociation was used as a robustness check. We first tested whether disorganized attachment predicts identity dissociation. Identity dissociation was modeled as a function of centered disorganized attachment:

**H1**. Disorganization → Identity dissociation

$$\text{log}\left(E\left[{\text{Identity}}_{i}\right]\right)=\beta_{0}+\beta_{1}{\text{disorganized}}_{\mathrm{c,i}}$$

Next, we tested whether childhood maltreatment predicts disorganized attachment. Disorganization was modeled as a function of centered childhood maltreatment:

**H2**. Childhood Maltreatment → Disorganization

$$\text{log}\left(E\left[{\text{disorgnized}}_{i}\right]\right)=\gamma_{0}+\gamma_{1}{\text{CTQ}}_{c,i}$$

These models establish the core pathway from trauma to disorganization to identity dissociation. We then tested two competing “locations” of genetic moderation along the childhood maltreatment → disorganized attachment → identity dissociation pathway. The first model, termed formation-level moderation model, assumes that *FKBP5* CATT moderates the association between childhood maltreatment and disorganized attachment. Disorganization was modeled as a function of centered childhood maltreatment, CATT status, and their interaction:

**H3a**. Formation-level moderation (Childhood Maltreatment × CATT → Disorganization)

$$\text{log}\left(E\left[{\text{disorganized}}_{i}\right]\right)=\gamma_{0}+\gamma_{1}{\text{CTQ}}_{c,i}+\gamma_{2}{\text{CATT}}_{i}+\gamma_{3}\left({\text{CTQ}}_{c,i}\times {\text{CATT}}_{i}\right)$$

The second model, termed the translation-level moderation model, assumes that *FKBP5* CATT moderates the association between disorganized attachment and identity dissociation, controlling for childhood maltreatment. Identity dissociation was modeled as a function of centered disorganization, centered childhood maltreatment, CATT status, and their interaction:

**H3b**. Translation-level moderation (Disorganization × CATT → Identity dissociation)

$$\text{log}\left(E\left[{\text{Identity}}_{\text{i}}\right]\right)=\beta_{0}\beta_{1}{\text{disorganized}}_{c,i}\beta_{2}{\text{CTQ}}_{c,i}\;+\beta_{3}{\text{CATT}}_{\text{i}}\beta_{4}\left({\text{disorganized}}_{c,i}\times {\text{CATT}}_{i}\right)$$

Relative support for the two moderation locations was evaluated using Bayesian Information Criterion (BIC), with lower BIC indicating better relative fit. For the significant H3b interaction, simple slopes analyses estimated the association between disorganized attachment and identity dissociation separately for CATT non-carriers and carriers. Predicted values on the response scale were derived using the *emmeans* package and visualized as regression curves with 95 % confidence bands.

Nonparametric bootstrap analyses with 2000 resamples were conducted for both H3a and H3b, extracting the interaction coefficient in each resample and computing 95 % percentile and BC*a* confidence intervals. As an additional check, H3b was re-estimated using OLS regression on log-transformed identity dissociation log(*Y*) with robust HC3 standard errors. B-spline terms (up to 3 df) were optionally included to model potential non-linear effects of disorganization on identity dissociation.

## 3. Results

### 3.1. Descriptive statistics

The analytic sample comprised 310 trauma-exposed adults with complete data across all key measures. Regarding the genetic moderator, 186 participants (60.0 %) carried at least one *FKBP5* CATT haplotype (carriers), and 124 participants (40.0 %) were non-carriers. The means and standard deviations of the study variables, along with their intercorrelations, can be found in Table 2.

Following our analytic strategy, we first tested the basic attachment-based pathway from childhood maltreatment to identity dissociation via disorganized attachment (H1 and H2). We then examined whether *FKBP5* CATT moderated this pathway, focusing on two competing models: moderation at the level of childhood maltreatment → disorganization (formation model, H3a) versus disorganization → identity dissociation (translation model, H3b).

### 3.2. Associations between trauma, disorganization, and identity dissociation (H1 and H2)

In line with attachment-based models of dissociation, disorganized attachment was positively associated with identity dissociation (H1). In a Gamma GLM predicting identity dissociation from centered disorganization, higher disorganization significantly increased expected identity dissociation (*β* = 0.143, SE = 0.029, *t* = 4.90, *p* < .001; see Table S1). Results were highly similar when using HC3 robust standard errors (*β* = 0.143, SE = 0.037, *t* = 3.83, *p* < .001; robust estimates shown in the right column of Table S1 in the supplement).

Childhood maltreatment was also associated with disorganized attachment (H2). In a Gamma GLM predicting disorganization from centered CTQ total scores, higher trauma significantly predicted higher disorganization (*β* =0.0074, SE = 0.0016, *t* = 4.68, *p* <.001; Table S2 in the supplement). Again, this effect remained virtually unchanged when using robust standard errors (*β* = 0.0074, SE = 0.0017, *t* = 4.46, *p* < .001; robust estimates in Table S2 in the supplement). Together, these results are consistent with the hypothesized sequential pathway, suggesting that childhood maltreatment is significantly linked to higher disorganized attachment, which in turn is associated with higher identity dissociation. While these findings support the theoretical model, the current analysis only confirms the presence of these associations between all variables in the hypothesized chain.

### 3.3. Moderation by FKBP5 CATT: formation vs. translation (H3a vs. H3b)

#### 3.3.1. Formation model

We next investigated where along this pathway *FKBP5* CATT acts as a moderator. In the formation model (H3a), disorganized attachment was regressed on childhood maltreatment, CATT status, and their interaction. Childhood maltreatment remained a significant predictor of disorganization (*β* = 0.0102, SE = 0.0028, *t* = 3.62, *p* < .001), but the childhood maltreatment × CATT interaction was non-significant (*β* = −0.0044, SE = 0.0034, *t* = −1.28, *p* =.20; Table S3 in the supplement). The corresponding robust model yielded the same pattern, with a slightly larger standard error for the interaction term (*β* = −0.0044, SE = 0.0037, *t* = −1.18, *p* = .24; robust estimates in Table S3 in the supplement). Thus, we found no evidence that *FKBP5* CATT alters the extent to which childhood maltreatment exposure leads to disorganized attachment.

#### 3.3.2. Translation model

In the translation model (H3b), identity dissociation was regressed on childhood maltreatment, disorganized attachment, CATT status, and the Disorganization × CATT interaction. Here, the interaction significantly predicted identity dissociation (*β* = 0.133, SE = 0.054, *t* = 2.46, *p* = .014; Table S4 in the supplement). In the same model, higher childhood maltreatment remained associated with higher identity dissociation (*β* = 0.0043, SE = 0.0011, *t* = 3.93, *p* < .001), and CATT carriers showed higher overall levels of identity dissociation than non-carriers (*β* = 0.087, SE = 0.040, *t* = 2.18, *p* = .029). The main effect of disorganization was small and non-significant (*β* = 0.020, SE = 0.043, *t* = 0.45, *p* = .65), consistent with the presence of a crossover interaction. Robust estimates again showed the same pattern, with the interaction remaining statistically significant (*β* = 0.133, SE = 0.060, *t* = 2.22, *p* = .027; Table S4 in the supplement, robust column).

#### 3.3.3. Model fit and bootstrap confidence intervals

Model comparison based on BIC favored the translation model (H3b) over the formation model (H3a). The H3b model predicting identity dissociation yielded a BIC of 231.88, whereas the H3a model predicting disorganization yielded a BIC of 497.18. Although these models have different outcomes and cannot be directly compared via likelihood-ratio tests, the large BIC difference is consistent with the impression that moderation is present for the disorganization-dissociation link but not for the trauma-disorganization link.

Bootstrap analyses further supported this pattern. For the Disorganization × CATT interaction (H3b), the 95 % percentile confidence interval ranged from 0.0212 to 0.2477, and the 95 % BC*a* interval ranged from 0.0233 to 0.2519, both excluding zero. In contrast, for the Trauma × CATT interaction (H3a), the 95 % percentile interval ranged from −0.0112 to 0.0030, and the 95 % BC*a* interval ranged from −0.0113 to 0.0028, both including zero. Thus, bootstrap results indicate robust evidence for the Disorganization × CATT interaction and no evidence for a childhood maltreatment × CATT interaction.

#### 3.3.4. Simple slopes and effect-size interpretation

**Fig. 2.**
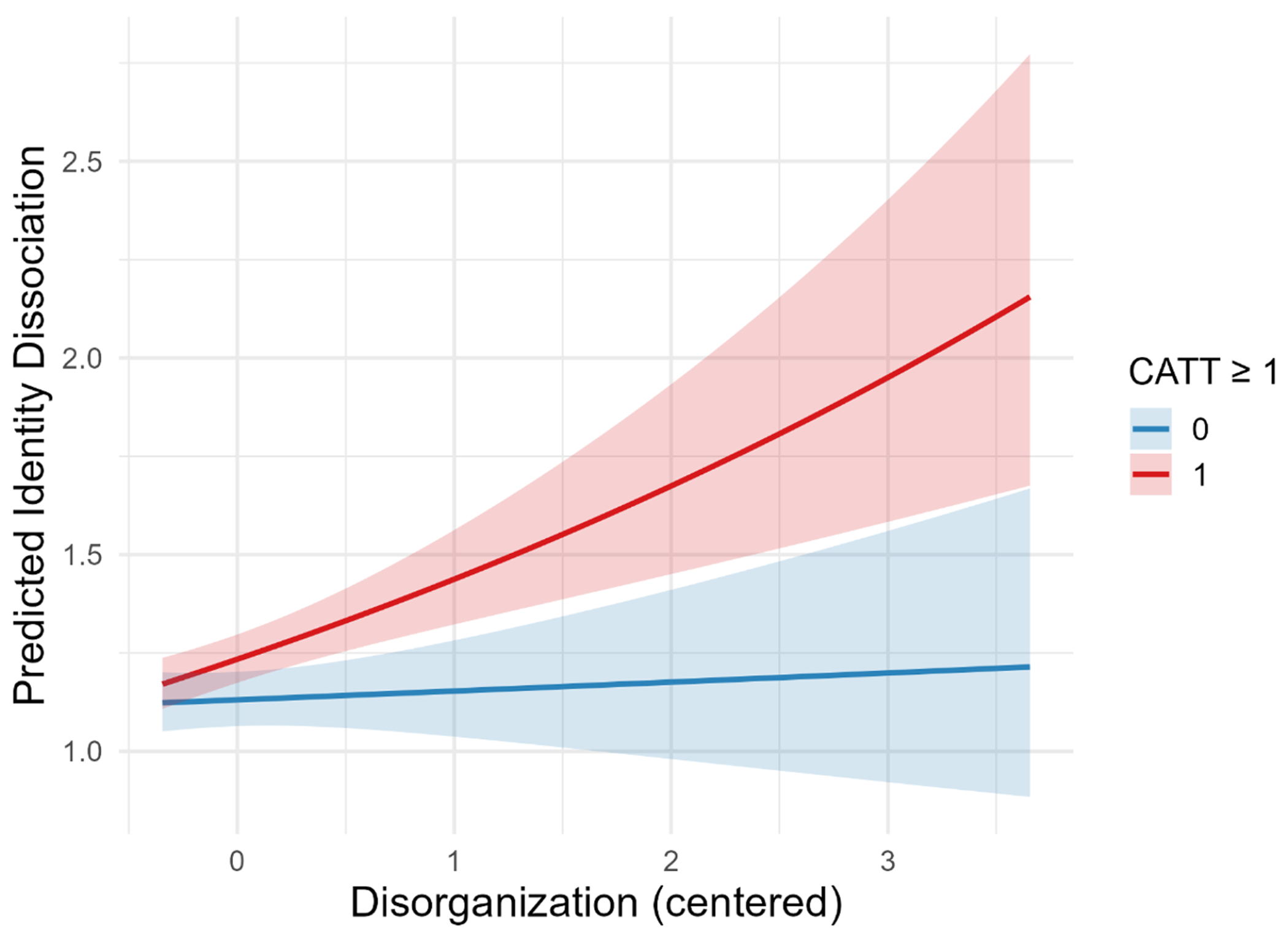
Moderating Effect of *FKBP5* CATT Haplotype on the Link between Disorganized Attachment and Identity Dissociation.

Simple-slopes analyses further clarified the nature of the Disorganization × CATT interaction. Among CATT non-carriers, disorganized attachment did not significantly predict identity dissociation (*β* = 0.02, SE = 0.04, *t* = 0.45, *p* = .65), corresponding to an estimated 1.97 % increase in expected identity dissociation per one-unit increase in disorganization. However, among CATT carriers, the slope was markedly stronger and statistically significant (*β* = 0.15, SE = 0.03, *t* = 4.43, *p* < .001), corresponding to an estimated 16.48 % increase in expected identity dissociation per one-unit increase in disorganization. Predicted values on the response scale (Fig. 2) showed that identity dissociation increased steeply with disorganized attachment among CATT carriers, whereas the corresponding curve for non-carriers was comparatively flat across the observed range of disorganization scores. This pattern strongly supports the Translation Hypothesis. Specifically, the significant and steeper slope for CATT carriers demonstrates that the *FKBP5* risk haplotype does not influence the formation of disorganized attachment (which would require a moderation effect at the previous step) but rather amplifies the translation or progression of pre-existing attachment disorganization into severe identity dissociation. This difference in slopes is the direct empirical evidence of the CATT haplotype’s role in amplifying the failure of integration *after* the initial attachment disruption.

#### 3.3.5. Alternative modeling check

To examine whether the interaction pattern depended on the Gamma specification, we re-estimated the H3b model using a linear regression of log-transformed identity dissociation log(*Y*) with HC3 robust standard errors. In this model, the Disorganization × CATT interaction remained in the same direction and approached conventional significance (*β* = 0.100, *SE* = 0.057, *t* = 1.75, *p* = .081). The main effects of childhood maltreatment and CATT status remained significant. Although somewhat attenuated in this more conservative specification, the pattern is consistent with the primary Gamma-model results, supporting the robustness of the interaction.

## 4. Discussion

This study examined where along a *trauma* → *attachment* → *dissociation* pathway the *FKBP5* CATT gene-environment effect on identity dissociation operates. Consistent with attachment-based theories, childhood maltreatment was linked to disorganized attachment, which in turn predicted elevated identity dissociation. We compared two potential loci of genetic moderation: (a) the trauma-disorganization link (formation hypothesis) and (b) the disorganization-identity dissociation link (translation hypothesis).

Three main findings emerged. *FKBP5* CATT did not significantly moderate the trauma-disorganized attachment association, suggesting it does not primarily influence the formation of disorganized attachment. In contrast, *FKBP5* CATT significantly moderated the disorganized attachment-identity dissociation association. Simple slopes indicated that disorganized attachment predicted identity dissociation only among CATT carriers. In sum, these results suggest that *FKBP5* CATT amplifies the *translation* of disorganized attachment into identity dissociation. It does not act in its initial *formation*.

These findings support attachment-based models of dissociation, which posit disorganized attachment as a proximal psychological vulnerability for dissociative outcomes. Early experiences with frightened or frightening caregivers foster conflicting internal working models of self and caregiver, producing disorganization (Liotti, 2004). Under ongoing stress, these unresolved representations may manifest as segmented or poorly integrated self-states (Lyons-Ruth et al., 2006; Şar et al., 2017).

Our results extend this model by suggesting genetic modulation of the disorganization-dissociation transition. Among *FKBP5* CATT carriers, disorganized attachment constitutes a high-risk state for identity dissociation. Non-carriers with similar levels of disorganization are less likely to exhibit severe identity fragmentation. Thus, *FKBP5* CATT may act as an ”amplifier” which increases the likelihood that disorganization translates into a failure of identity integration. Given *FKBP5*’s role in the regulation of stress response, disorganized attachment states in carriers may be more tightly coupled to stress-reactive neurobiological cascades and may impair the development of reflective processing of self and relational experiences (Halldorsdottir et al., 2019; Malekpour et al., 2023).

Our model is aligned with the perspective of developmental psychopathology, which suggests that the pathway from childhood maltreatment to identity dissociation is tied to critical, stress-sensitive periods of neurobiological and psychological integration (Kearney and Lanius, 2022). Empirical longitudinal and retrospective data support the timing of our proposed sequence. For example, disorganized attachment patterns established as early as 12–18 months have been identified as robust predictors of dissociative symptoms in late adolescence and early adulthood (Ogawa et al., 1997). Although dissociative-like phenomena (e.g., imaginary companions) may be a normal part of preschool development, these behaviors appear to become pathologically “fixed” following severe early trauma (Bernier et al., 2013). Consequently, psychopathology may only become apparent when individuals reach adolescence or young adulthood, even if underlying symptoms were present earlier but were not viewed as overtly pathological within a developmental context. Recent evidence supports a “sensitive period” model of maltreatment. While post-traumatic stress disorder (PTSD) severity often follows a dose-response relationship regardless of the timing of trauma, severe dissociative symptoms are specifically predicted by neglect and abuse occurring during early childhood, particularly around ages 4–5 (Schalinski et al., 2016; Yehuda et al., 1997). This suggests that the transition from trauma to dissociative phenomena in our model is likely rooted in the preschool years; a sensitive period when neurobiological systems underlying self-integration are particularly plastic (Kearney and Lanius, 2022). We propose that identity dissociation reflects a primary failure of identity integration during these formative stages. While this may manifest later as a subjective “fragmented self”, it represents a developmental trajectory where a stable, integrated self-structure was *never fully established*. In contrast, trauma sustained in adulthood encounters a relatively stable and consolidated psychological architecture. While adult-onset trauma frequently precipitates PTSD or dissociative detachment (e.g., depersonalization and derealization), it lacks the developmental leverage to induce the structural identity fragmentation typically seen following early-life relational adversity (Morgan et al., 2001).

The CATT haplotype is associated with upregulation of the *FKBP*5-gene, which in turn is linked to dysfunction of the HPA axis feedback loop (Binder et al., 2004). In addition, *FKBP*5 is involved in many other signaling pathways that contribute to the stress response to negative life events, such as cell proliferation, large-scale brain network connectivity, autophagy, and the autoimmune system (Halldorsdottir et al., 2019; Hwang et al., 2025; Malekpour et al., 2023; Marroquin Rivera and Labonté, 2025; Zhang et al., 2020). The CATT haplotype has already been linked to increased anxiety, impaired extinction learning, poorer self-regulation and reduced stress coping (Halldorsdottir et al., 2019; Malekpour et al., 2023; Matosin et al., 2018). During the formative years (from birth to around the age of nine), the biopsychological architecture is very plastic. Chronic traumatic stress during this developmental phase, combined with a disorganized attachment style and increased molecular stress sensitivity due to the *FKBP*5-CATT variant, could contribute to the absence of the necessary biopsychological prerequisites for the development of a unified identity, such as undisturbed functioning of the hippocampus, frontal cortex, and amygdala (Kearney and Lanius, 2024; Roozendaal et al., 2001; Sapolsky, 2015).

These findings highlight the importance of integrating genetic and attachment-based mechanisms in models of complex trauma-related psychopathology. Rather than treating genetic risk and disorganized attachment as independent predictors, our results suggest that genetic vulnerability may operate as a transdiagnostic endophenotype specifically *within* disorganized attachment states.

Clinically, disorganized attachment emerges as a critical yet often neglected assessment and intervention target for individuals with histories of childhood maltreatment and dissociative symptoms (Bryant, 2023; Mikulincer and Shaver, 2012), particularly when biological vulnerability is present. While genetic testing is not routine for the foreseeable future, the findings suggest that individuals with pronounced disorganization may differ in their risk for identity dissociation based on underlying biological sensitivity.

For individuals presenting with complex sequelae of trauma, including severe dissociation, psychotic symptoms, and negative self-concept, bona fide trauma-focused therapies remain the treatment of choice (Banz et al., 2022; Bloomfield et al., 2020; Ehring et al., 2014; Hoeboer et al., 2020; Kip et al., 2025; Kratzer et al., 2023; Maercker et al., 2022; Schaug et al., 2025). Consequently, rather than replacing these established interventions, attachment-informed approaches should be integrated to optimize outcomes. Specifically, attachment-focused interventions that enhance mentalization, reflective functioning, and integrative narrative processing (e.g., Smits et al., 2024) may be critical for the high-risk disorganized/CATT group. These approaches could serve to buffer the translation of disorganized attachment into rigidly segmented self-states and identity dissociation, ensuring that the necessary trauma processing is conducted within a safe relational framework that explicitly addresses attachment trauma and promotes a coherent sense of self.

Several limitations temper these conclusions. Our cross-sectional design precludes causal inference; future longitudinal studies are needed to confirm the proposed sequence and the locus of genetic moderation. Due to a lack of psychometric or genetic data, only about three percent of the original population could be included in the study. The small sample size, which consists of African Americans from urban backgrounds (a strength in terms of representativeness), could limit the interpretability and generalizability of the results. Future replication of the results in larger and more diverse populations is necessary.

Statistical power constrained more complex modeling, and we dichotomized the CATT haplotype; future work should explore finer-grained genetic coding, polygenic indices, or additional *FKBP5* variants, and use a structural equation modelling framework. A further, crucial limitation is that we cannot reliably assess caregiving behavior in early infancy, and our use of the CTQ provides only a retrospective proxy with limited validity as a specific precursor of disorganized attachment. This constrains the precision with which we can model the developmental pathway from early caregiving to later attachment disorganization. Finally, we did not directly measure HPA-axis function, so our proposed stress-reactive mechanisms remain speculative.

Future research should (a) employ longitudinal designs to track the emergence and course of disorganized attachment and dissociation, (b) incorporate biomarkers of HPA-axis and related systems, and (c) examine whether attachment-focused interventions are particularly effective in individuals at elevated biological risk. This may further clarify when disorganized attachment serves as a ”tipping point” and when it remains a marker of adversity without progressing to identity dissociation.

### 4.1. Concluding remarks

Identifying the importance of childhood maltreatment and disorganized attachment in the development of identity dissociation provides two broad targets for psychological intervention. However, by providing evidence for the Translation Hypothesis, this study refines the therapeutic focus. Our finding indicates that the *FKBP5* CATT haplotype does not determine whether disorganized attachment forms but rather dictates whether that pre-existing attachment failure translates into the severe outcome of identity dissociation. This highlights a critical, genetically-influenced post-attachment failure period where interventions are most needed. Therefore, our results provide a rationale for targeted, early intervention for trauma-exposed young people and young adults presenting with disorganized attachment, specifically aiming to bolster ”integrative capacity” (Pierre Janet as cited in Van der Hart and Rydberg, 2019) and prevent the progression to severe identity dissociation.

## Supplementary Material

1

Supplementary material associated with this article can be found, in the online version, at doi:10.1016/j.psychres.2026.117079.

## Figures and Tables

**Table 1:** Demographic characteristics.

	*N*	%
***Relationship status***		
Single, never married	180	58.1
Married	32	10.3
Divorced	61	19.7
Separated	15	4.8
Widowed	7	2.3
Domestic Partner	15	4.8
***Education***		
Less than twelfth grade	64	20.6
12th Grade/High School Graduate	97	31.3
General Educational Development	14	4.5
Some college or technical school	73	23.5
Technical school graduate	23	7.4
College graduate	31	10
Graduate school	8	2.6
***Employment***		
No	220	71
Yes	89	28.7
N/A	1	0.3
***Disability***		
No	248	80
Yes	61	19.7
N/A	1	0.3

**Table 2:** Means, standard deviations, and correlations with 95 %-confidence intervals.

Variable	*M*	*SD*	1	2	3	4	5	6	7
1. CTQ Emotional Abuse	9.20	5.09							
2. CTQ Sexual Abuse	8.25	5.41	.57** [.49, 0.64]						
3. CTQ Physical Abuse	8.03	4.10	.71** [.66, 0.77]	.48** [.39, 0.56]					
4. CTQ Physical Neglect	6.65	3.06	.62** [.55, 0.69]	.49** [.40, 0.57]	.56** [.48, 0.64]				
5. CTQ Emotional Neglect	9.54	5.07	.74** [.68, 0.78]	.50** [.41, 0.58]	.58** [.50, 0.65]	.65** [.58, 0.71]			
6. MDI Identity Dissociation	1.21	0.51	.30** [.19, 0.39]	.20** [.09, 0.30]	.17** [.06, 0.27]	.23** [.12, 0.34]	.26** [.15, 0.36]		
7. AAPQ Disorganized Prototype	1.35	0.74	.28** [.18, 0.38]	.22** [.11, 0.32]	.19** [.08, 0.30]	.15** [.04, 0.26]	.20** [.09, 0.31]	.27** [.16, 0.37]	
8. CATT Haplotyples	0.72	0.66	.11 [−.01, 0.21]	.08 [−.03, 0.19]	.11 [−.00, 0.22]	.01 [−.10, 0.13]	.08 [−.03, 0.19]	.16** [.05, 0.27]	.10 [−.01, 0.21]

*Note. M* and *SD* are used to represent mean and standard deviation, respectively. Values in square brackets indicate the 95 % confidence interval for each correlation.

* indicates *p* < .05.

** indicates *p* < .01.

## Data Availability

The datasets used and/or analysed during the current study are available from Abigail Powers Lott (Co-Director, Grady Trauma Project) on reasonable request.
