# Supplementary material for: Disorganized attachment and identity dissociation: FKBP5 CATT as molecular moderator
Source: Psychiatry Res. Author manuscript; Available in PMC 2026 Jun 25. (PMC13294002; doi:10.1016/j.psychres.2026.117079)
Supplement: 1 [file NIHMS2179286-supplement-1.docx]

**Supplement**

Table s1. H1: Disorganized Attachment → Identity Dissociation

| Parameter | Estimate | SE | t | p | 95% CI | Estimate (robust) | SE (robust) | t (robust) | p (robust) | 95% CI (robust) |
| --- | --- | --- | --- | --- | --- | --- | --- | --- | --- | --- |
| Intercept | 0.182 | 0.02169 | 8.41 | <.001 | 0.140-0.225 | 0.182 | 0.02181 | 8.37 | <.001 | 0.140-0.225 |
| Disorganized | 0.143 | 0.02929 | 4.90 | <.001 | 0.085-0.203 | 0.143 | 0.03741 | 3.83 | <.001 | 0.070-0.217 |

Table s2. H2: Childhood Maltreatment → Disorganized Attachment

| Parameter | Estimate | SE | t | p | 95% CI | Estimate (robust) | SE (robust) | t (robust) | p (robust) | 95% CI (robust) |
| --- | --- | --- | --- | --- | --- | --- | --- | --- | --- | --- |
| Intercept | 0.287 | 0.02943 | 9.75 | <.001 | 0.230-0.345 | 0.287 | 0.02953 | 9.71 | <.001 | 0.229-0.345 |
| CTQ | 0.00739 | 0.00158 | 4.68 | <.001 | 0.0043-0.0106 | 0.00739 | 0.00166 | 4.45 | <.001 | 0.0041-0.0106 |

Table s3. H3a: Formation Model: Trauma × CATT → Disorganized Attachment

| Parameter | Estimate | SE | t | p | 95% CI | Estimate (robust) | SE (robust) | t (robust) | p (robust) | 95% CI (robust) |
| --- | --- | --- | --- | --- | --- | --- | --- | --- | --- | --- |
| Intercept | 0.277 | 0.04679 | 5.93 | <.001 | 0.187-0.370 | 0.277 | 0.04665 | 5.94 | <.001 | 0.185-0.369 |
| CTQ | 0.01015 | 0.00281 | 3.62 | <.001 | 0.0049-0.0156 | 0.01015 | 0.00316 | 3.21 | .001 | 0.0039-0.0164 |
| CATT | 0.02040 | 0.06028 | 0.34 | .735 | -0.098-0.138 | 0.02040 | 0.06066 | 0.34 | .737 | -0.099-0.140 |
| CTQ x CATT | -0.00437 | 0.00340 | -1.28 | .199 | -0.0111-0.0022 | -0.00437 | 0.00370 | -1.18 | .239 | -0.0116-0.0029 |

Table s4. H3b: Translation Model: Disorganized Attachment × CATT → Identity Dissociation

| Parameter | Estimate | SE | t | p | 95% CI | Estimate (robust) | SE (robust) | t (robust) | p (robust) | 95% CI (robust) |
| --- | --- | --- | --- | --- | --- | --- | --- | --- | --- | --- |
| Intercept | 0.1231 | 0.03102 | 3.97 | <.001 | 0.063-0.185 | 0.1231 | 0.02575 | 4.78 | <.001 | 0.072-0.174 |
| CTQ | 0.00430 | 0.00109 | 3.93 | <.001 | 0.0021-0.0065 | 0.00430 | 0.00145 | 2.97 | .003 | 0.0015-0.0071 |
| Disorganized | 0.0195 | 0.04308 | 0.45 | .651 | -0.063-0.106 | 0.0195 | 0.03714 | 0.53 | .600 | -0.054-0.093 |
| CATT | 0.0873 | 0.04009 | 2.18 | .029 | 0.008-0.166 | 0.0873 | 0.03712 | 2.35 | .019 | 0.014-0.160 |
| Disorganized x CATT | 0.1330 | 0.05419 | 2.46 | .014 | 0.025-0.240 | 0.1330 | 0.06003 | 2.22 | .027 | 0.015-0.251 |
